# Resultant Incidence Angle: A Unique Criterion for Controlling the Inclined Columnar Nanostructure of Metallic Films

**DOI:** 10.3390/nano15080620

**Published:** 2025-04-18

**Authors:** Aurélien Besnard, Hamidreza Gerami, Marina Raschetti, Nicolas Martin

**Affiliations:** 1 Université Marie et Louis Pasteur, SUPMICROTECH, CNRS, Institut FEMTO-ST, F-25000 Besançon, France; hamidreza.gerami@femto-st.fr (H.G.); nicolas.martin@femto-st.fr (N.M.); 2 Université Marie et Louis Pasteur, CNRS, Institut FEMTO-ST, F-25000 Besançon, France; marina.raschetti@femto-st.fr

**Keywords:** Glancing Angle Deposition, transport simulation, prediction of the column tilt angle

## Abstract

The original Glancing Angle Deposition (GLAD) technique was developed using the evaporation process, i.e., in high vacuum, with a nearly punctual source, and with the substrate aligned with the source axis. In this specific case, the substrate tilt angle can be assumed to be equal to the impinging incidence angle of evaporated atoms. With the sputtering process, the deposition pressure is higher, sources are larger, and substrates are not intrinsically aligned with the source. As a result, deviations from the growth models applied for evaporation are reported, and the substrate tilt angle is no longer relevant for describing the impinging atomic flux. To control the inclined nanostructure of metallic films, a relevant description of the atomic flux is required, applicable across all deposition configurations. In this work, transport simulation is used to determine the resultant incidence angle, a unique criterion relevant to each specific deposition condition. The different representations of the flux are described and discussed, and some typical examples of the resultant angles are presented. Ten elements are investigated: three hcp transition metals (Ti, Zr, and Hf), six bcc transition metals (V, Nb, Ta, Cr, Mo, and W), and one fcc post-transition metal (Al).

## 1. Introduction

Engineering material structures on an increasingly smaller scale presents a challenging task for developing original devices at the sub-micrometer scale. Although the top-down approach has rapidly exhibited some limitations in nano-fabrication due to the restricted dimensions of the sculpting tools, the bottom-up strategy has gained importance as the correct and ideal method for producing new and original designs at the micro- and nanoscale [1]. The creation of an atomic source, the transportation of atoms from the source to the substrate surface, and the condensation–nucleation phenomena are commonly regarded as the fundamental stages for depositing thin films of various compounds. These three stages are thus decisive for the final shape and features of the as-deposited film, and they strongly influence the ability to tune the film properties through physical structuring. As a result, some vacuum deposition techniques have been thoroughly developed to fabricate novel nanostructured columnar architectures [2,3,4,5]. In recent decades, a strong emphasis has been placed on the Glancing Angle Deposition (GLAD) process and, in a more comprehensive way, on oblique angle deposition (OAD), which is now renowned as a key enabler for structuring thin films [6,7,8,9,10]. This process allows for the fabrication of a wide range of nanostructures like tilted columns, zigzags, helices and so on, which are now typically reported in the literature for metals, semiconductors, and dielectrics [11,12,13,14,15,16].

These resulting architectures are formed due to the ballistic shadowing effect at the atomic scale, which can be managed by means of precise control of the substrate motion (the incidence of the particle flux and substrate rotation). Regardless of the final structure, the directional growth allows for the production of a film morphology consisting of inclined columns with a tilted angle, β, defined as the angle between the substrate normal and the column center axis. Until now, many analytical equations have been suggested that provide more or less accurate connections between the incidence angle, α, and the column tilt angle, β (e.g., Tangent rule, Tait’s rule, fan-out model) [17,18,19,20,21,22]. However, these suggested equations are not derived from physical models, and the predicted column angles exhibit some inconsistencies with experimental measurements. These discrepancies are mainly attributed to certain simplifying assumptions, such as a purely ballistic character of the impinging particles or the significant role of the crystalline structure of the deposited material [23]. In addition, these theoretical analyses ignore the strong differences between the simple evaporation configuration (low pressure, low energy, small source dimensions) and the more geometrically complex sputtering processes (higher pressure, higher energy of particles, larger sputtering sources).

**Figure 1:**
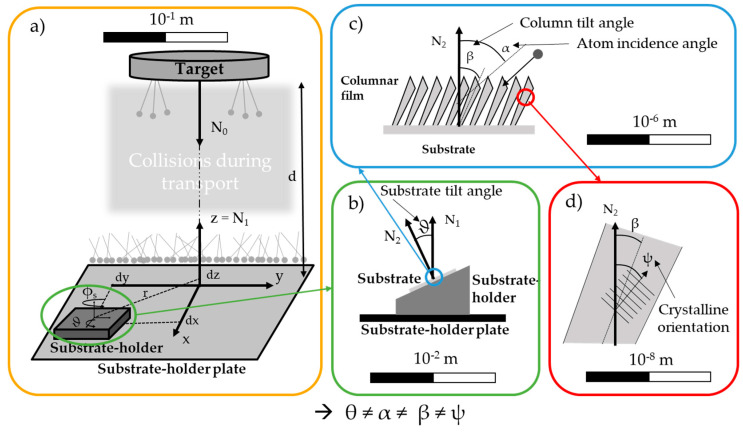
Schemes of the angles involved in oblique angle deposition at different scales: (**a**) chamber scale; (**b**) substrate holder scale; (**c**) film scale; and (**d**) column scale. d is the distance from the target to the substrate holder, (dx, dy, and dz) are the coordinates of the substrate holder on the plate, r is the distance between the substrate and the target axis in the plane of the plate, N_0_ is the normal to the target, N_1_ is the normal to the substrate holder plate, N_2_ is the normal to the substrate, ϑ is the substrate tilt angle, α is the incidence angle, ϕ is the azimuthal angle, β is the column tilt angle, and ψ is the crystallite tilt angle.

Consequently, a better description of the sputtered fluxes, and thus more reliable predictive relationships between α and β angles, emerges as a scientific motivation and a technological requirement for structuring thin films by OAD. In this context, a comprehensive description of the different angles involved, from nanometric to centimetric scales, is necessary (Figure 1).

Four scales are then defined with different geometrical parameters as follows:

- Chamber scale: Three reference frames are set. The reference frame of the target is located at the center of the target, with the z-axis set as normal N_0_. The reference frame of the substrate holder plate is aligned with N_0_ at a distance d from the target reference frame, with the z-axis set as normal N_1_. The reference frame of the substrate holder has a z-axis collinear to N_1_, with offsets ((dx, dy, dz) or (r, dz)) and rotations (the substrate azimuthal angle, ϕ_s_, about the *z*-axis and the substrate tilt angle, ϑ, about another axis). A geometrical angle, ϑ_geo_, can be defined between the first and third reference frames and added to the tilt.
- Substrate scale: ϑ (or ϑ_geo_) represents the substrate tilt angle previously introduced. It is defined as the angle between the substrate normal N_2_ and the normal of the substrate holder plate N_1_. This angle serves only a geometric purpose and is primarily useful for sample identification. It is merely one of many factors affecting the deposition conditions. ϑ is determined by geometric measurements of the deposition system.
- Film scale: α represents the atomic incidence angle, and ϕ represents the atomic azimuthal angle. These angles define the final trajectory of each atom impinging on the substrate. The incidence angle, α, is out of the substrate plane and is defined relative to the substrate normal N_2_. The azimuthal angle, ϕ, lies in the substrate plane and is defined relative to the x- or y-axis. α and ϕ can only be determined through transport simulations, as they depend on the geometrical configuration of the system and the scattering of the flux due to the collisions of the metallic species with the gas atoms, which is associated with the mean free path. β is the column tilt angle, defined relative to the substrate normal N_2_. As a flux azimuthal angle is used, it could also be logical to take into account a column azimuthal angle. Most of the time, it is commonly assumed that this angle is aligned with one of the axes of the substrate. Indeed, β is experimentally obtained from SEM cross-section images after sample fracture.
- Columnar microstructure level: ψ represents the crystalline tilt angle and is defined relative to the substrate normal, N_2_. ψ is determined from XRD pole figures.

The substrate tilt angle, ϑ, the atomic incidence angle, α, the column tilt angle, β, and the crystalline tilt angle, ψ, are generally not equal. ϑ and α can be the same in some specific cases, e.g., in the case of the evaporation process assuming a pure ballistic regime of particles impinging on the substrate, but they should not be confused, as their nature is very different. The relationship between these angles can be modeled with analytical laws. However, as other physical phenomena (associated with the energy of the system) are involved, the relationships are not straightforward. Moreover, it is primarily important to have a correct description of the flux before discussing this relationship.

In this paper, we report on the different representations of the metallic flux impinging on a given surface, obtained through simulation based on the manner of processing the data. This simulation concerns only the geometrical description of the flux, i.e., the atomic incidence angle, α, and the azimuthal angle, ϕ. Here, the substrate tilt angle, ϑ, is an input value, the column tilt angle, β, is an output value, and the crystalline tilt angle, ψ, is not discussed.

It is assumed that this geometrical aspect is the main contribution to the inclined columnar growth. Other contributions to growth, i.e., contamination, reactive gas, sticking coefficient, diffusion, crystallinity, material-dependent fan-out angle, substrate topography, and substrate temperature, undoubtedly influence hypotheses and require changes to this base. These contributions are primarily neglected and thus not discussed here, as they each depend on specific deposition conditions. They will be important when the relationship between the incidence angle, α, and the column tilt angle, β, is discussed. However, the argument regarding this relationship can only be analyzed if the incidence angle (x-axis) is relevant. This paper aims to provide this information.

Various representations of atomic flux are described and discussed, and some typical examples of the resultant angles are presented, concerning ten elements: three hcp (hexagonal close-packed) transition metals (Ti, Zr, and Hf), six bcc (body-centered cubic) transition metals (V, Nb, Ta, Cr, Mo, and W), and one fcc (faced-centered cubic) post-transition metal (Al). These elements come from independent studies that were chosen to cover a wide range of configurations. At first, the influence of the pressure, the position of the substrates, and the deposited element are studied in the simple OAD configuration. This approach is finally extended to a more complex process: OAD co-sputtering, which implements two tilted and separated atomic sources.

## 2. Materials and Methods

### 2.1. GLAD Configuration

**Figure 2:**
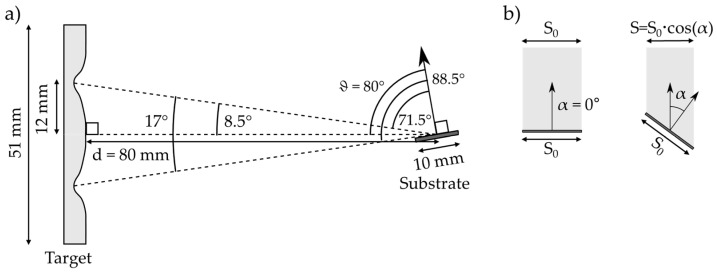
(**a**) Schemes of the GLAD configuration, (**b**) variation of the projected area, S, with the angle.

A classical GLAD configuration was chosen as a digital model case study. A substrate with dimensions of 1 × 1 × 0.1 cm was aligned with a 2” planar target at a distance d of 8 cm (center to center). The racetrack was obtained from experimental measurements of a used target. Titanium, as the deposited material, was chosen as an example. Any other metal would have been suitable. The substrate has a tilt angle, ϑ, of 80° (Figure 2a).

The substrate tilt angle, ϑ, is measured between the normal of the substrate and the line passing through the center of the target and the center of the substrate. This line also coincides, in this case, with the normal of the theoretical target surface. The maximum depth of the racetrack, which corresponds to the most probable sputtering area, is found at 12 mm from the center. The sputtering ring and the center of the substrate form a cone with an aperture of around 17°. Consequently, using a projection in the plane of the figure, the effective substrate inclination angle, ϑ, must be 71.5° (upper racetrack) with a small contribution from an inclination of 88.5° (lower racetrack). The respective contributions are determined using the “cosine law”, which rules the projection of the substrate surface in a plane perpendicular to the theoretical straight direction (Figure 2b). This gives an idea of the effective deposition rate, defined here as the number of atoms crossing this projected area and assumed to reach the substrate. This quantity is also related to the thickness of the films. Here, the difference is about 91% for ϑ = 71.5° (cos(71.5°) = 0.317, cos(80°) = 0.114, and cos(88.5°) = 0.026). These pure geometrical considerations will be tuned by scattering during the flight from the target to the substrate, which depends on the pressure, the initial ejection profile, and the shape of the racetrack.

Three working pressures were chosen: 0.1 Pa, 0.5 Pa, and 1.0 Pa. The low-pressure condition ensures few or no collisions between the target and the substrate (fewer than 1), resulting in a narrow, nearly ballistic flux. The high pressure implies a high number of collisions (~100) and necessarily a very diffuse flux. The medium pressure will create intermediate transport conditions (~27 collisions).

### 2.2. Numerical Conditions

The transport calculations are performed using SIMTRA (V2.2) [24]. In order to achieve sufficient accuracy in the following analyses, a large number of particles detected on the substrate is mandatory. In this work, to have enough points to ensure a relevant statistic, the objective was to detect a few hundred thousand. Specifically, 4.7 × 10^8^, 2.5 × 10^8^, and 1.8 × 10^8^ particles were sputtered, resulting in 5.3 × 10^5^, 5.3 × 10^5^, and 7.7 × 10^5^ detected particles for P = 0.1, 0.5, and 1.0 Pa, respectively.

**Figure 3:**
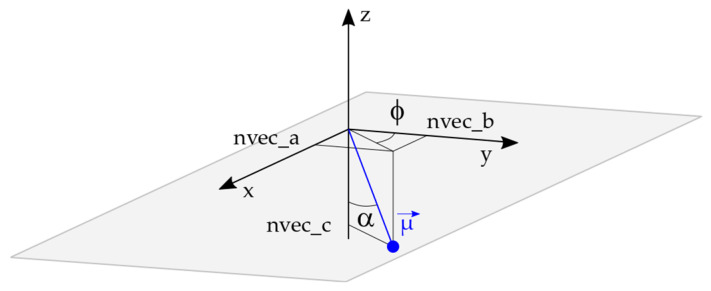
Description of the angles (α is the incidence angle, and ϕ is the azimuthal angle) and the coordinate of the unitary vector, $\vec{µ}$, which is composed of three coordinates (x = nvec_a, y = nvec_b, and z = nvec_c) in the substrate reference frame.

SIMTRA provides, among other parameters, the final direction of a particle crossing a specific surface. This direction is defined by a unitary vector composed of three coordinates (here in the local surface reference frame): x = nvec_a, y = nvec_b, and z = nvec_c. Starting from this vector, it is possible to calculate the two angles that will characterize the flux (Figure 3).

The incidence angle, α (out of the plane angle), is given by Equation (1) as follows:

α = −acos(nvec_c),(1)

The azimuthal angle, ϕ (in-plane angle), is given by Equation (2), relative to the *y*-axis, as follows:

ϕ = acos(−nvec_b/sin(nvec_c)),(2)

The azimuthal angle depends on the choice of the reference axis (x, −x, y, −y); a 90° phase shift can appear between two analyses if the formula is changed.

This vector, combined with the energy of the impinging atom, can be used in growth simulation code as Simul3D (V1.0) [25] or Virtual Coater© NASCAM (V4.8) [26]. In this work, Simul3D was used with a simulation box of 252 × 268 × 200 unit cells, which corresponds to a maximum number of atoms of 1.35 × 10^7^. The flux calculated by SIMTRA was used as the input, and diffusion was neglected, i.e., the simulation worked in a simple aggregation mode.

## 3. Simulation Results

For a given series of vectors, the following data treatments are possible in order to analyze the flux:

- To calculate α and ϕ and draw the distribution of the couple (α, ϕ);
- To calculate α and ϕ and draw their distributions independently;
- To determine the resultant vector (sum or average of each coordinate of the concerned series) and calculate the resultant angles, α_res_ and ϕ_res_. These two angles are the scalar criteria representing the flux.

These three treatments applied to the three deposition conditions are detailed and analyzed in the following section. It should be noted that the assumptions made in the SIMTRA model are potential sources of error in the flux calculation. Nevertheless, the three flux representations are independent of the code used for the transport simulation.

Table 1 provides a summary of the three considered working pressures at different angles describing the case study: the substrate tilt angle, ϑ, the three possible incidence angles, α, calculated from the SIMTRA results, and the column tilt angle, β, obtained from the Simul3D growth simulation.

**Figure 4:**
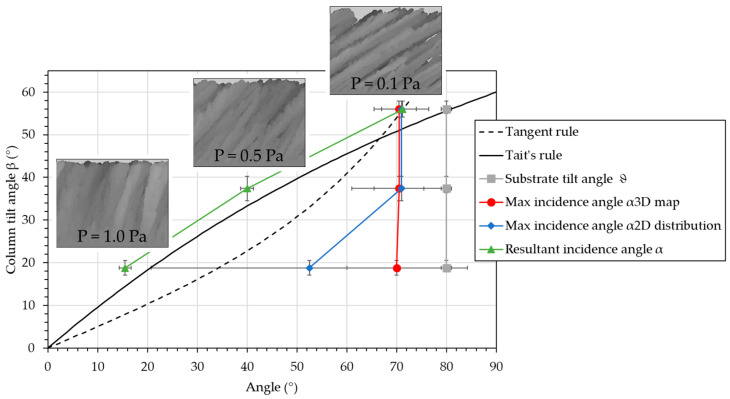
Column tilt angle, β, as a function of the different angles (the substrate tilt angle, ϑ, and the three possible incidence angles, α). The inclined microstructures from Simul3D are also presented. The Tangent rule and Tait’s rule are plotted as a guide for the eye.

Figure 4 presents the column tilt angle, β, as a function of the different angles (substrate tilt angle, ϑ, and the three possible incidence angles, α). The images from Simul3D representing the inclined microstructure are also presented.

The two classical rules are also presented on the graph, i.e., the “Tangent rule” [17] (Equation (3)) and “Tait’s rule” [18] (Equation (4)), expressed as follows:

(3) $$\tan\beta \;=\frac{\tan\alpha }{2}$$

(4) $$\beta =\alpha -\sin^{-1}\left(\frac{1-\cos\alpha }{2}\right)$$

It is observed that the column tilt angle, β, decreases with pressure, as expected due to the increase in scattering during transport. As explained, the substrate tilt angle is constant and consequently fails to predict the evolution of the column tilt angle, β. The maximum of the 3D distribution map is no longer relevant, as it remains mostly constant. With the maximum of the 2D distribution, an evolution similar to the Tangent rule can be estimated. However, the accuracy is poor (between 5° and 31.7°), and the deviation from the Tangent rule is high (between 5° and 25°). Consequently, the maximum of the 2D distribution cannot be considered a relevant and effective criterion. Using the resultant incidence angle, α, the evolution of Tait’s rule is followed with a deviation of less than 5°. It is worth noting that the column tilt angles are systematically higher than those of Tait’s rule. As it is obtained from a simple simulation, this value may be overestimated compared to a real deposition.

Among the three possible incidence angles that can be determined from the simulated dataset, only the resultant incidence angle is effective in predicting the evolution of the column tilt angle, β. The following section will discuss the meaning of the three incidence angles and explain why the resultant angle is the only effective criterion.

## 4. Discussion

This section details the three different data treatments available for analyzing the flux: the 3D distribution map of the couple (α, ϕ), the 2D distribution of the individual α and ϕ, and the resultant angles, α_res_ and ϕ_res_.

### 4.1. (α, ϕ) Distribution

**Figure 5:**
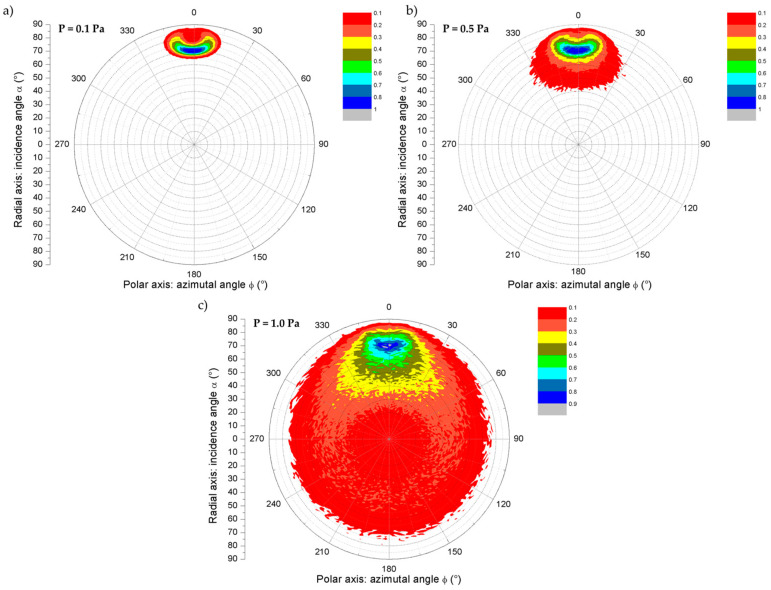
Three-dimensional (α, ϕ) distributions for the three working pressures: (**a**) 0.1 Pa, (**b**) 0.5 Pa, and (**c**) 1.0 Pa. The color scale represents the probability of an (α, ϕ) direction.

Figure 5 presents the 3D map of the (α, ϕ) distributions for the three working pressures: (a) 0.1 Pa, (b) 0.5 Pa, and (c) 1.0 Pa.

The probability was normalized to the maximum of each distribution, and ten probability ranges were used. This represents the probability for atoms in the flux to take a given (α, ϕ) direction. These representations are useful for providing a global overview of the flux. They are also used in 3D growth simulation. The disadvantage of these maps is that they require a large amount of data to be accurate, e.g., for a (α, ϕ) cell 2 × 5° in size, the map will have 3.24 × 10^3^ cells, and if a minimum of 100 atoms per cell is expected, then 3.24 × 10^5^ atoms detected on the substrate are needed. This will require a longer computation time (the computation time is linear with the number of atoms, as each atom is independent of the others) and will produce large datasets.

In all three maps presented in Figure 5, the highest probability (>0.8) is found around α = 70° ± 3 to 10° (Table 1) and ϕ = 0 ± 7.5°. The image of the target on the substrate is seen with an oval spot (with dimensions of α = [65, 85] and ϕ = [−15, +15]), and the upper racetrack (Figure 2) is observed as a bean-like spot. For 0.1 Pa, all deciles higher than 0.1 are within this spot, confirming that this case is nearly ballistic. At higher pressures, collisions during flight induce dispersion of the flux, and the deciles within the spot increase, i.e., higher than 0.4 for P = 0.5 Pa and higher than 0.6 for P = 1.0 Pa. For P = 0.5 Pa, all deciles higher than 0.1 are in the range α = [40, 85] and ϕ = [−30, +30], while for P = 1.0 Pa, deciles under 0.3 cover the full map. This means that a non-negligible number of atoms can come from the whole space with nearly any direction, especially from the back of the substrate. This will necessarily tend to decrease the column tilt angle.

However, the growth is an aggregation phenomenon, and the probability ranges within these maps are independent of one another. This explains why the maximum of the maps is not able to provide a complete description of the flux that can be used in column tilt angle predictions. Nevertheless, these maps are essential when they are employed in growth simulations where aggregation is performed, eventually with surface diffusion.

### 4.2. α. and ϕ Distributions

**Figure 6:**
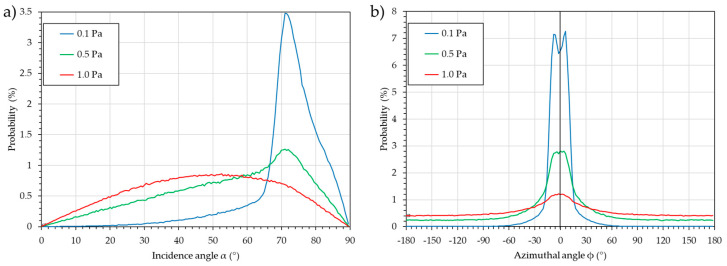
Two-dimensional distributions for the three working pressures (0.1, 0.5, and 1.0 Pa): (**a**) incidence angle, α, and (**b**) azimuthal angle, ϕ.

Figure 6 presents the most common representation of the flux, i.e., α and ϕ distributions obtained independently.

The probabilities are normalized to the number of atoms in the datasets, so that they are comparable. For these distributions, the number of data points required for accuracy is lower than in the previous case: for a cell of α = 2° or ϕ = 5° and 100 atoms per cell, 4.5 × 10^3^ and 7.2 × 10^3^ atoms are required for α and ϕ, respectively. Experience proves that 1.5 × 10^4^ to 2.0 × 10^4^ or more is better. Indeed, the greater the number of atoms, the better, as the computation time remains short and the dataset light.

An obvious influence of pressure is observed, with the distribution tending to be broader and the maximum probability decreasing. For the incidence angle, α, the influence of the upper racetrack is observed, with strong peaks at α = 71° ± 5° to 10° for P = 0.1 and P = 0.5 Pa and a slight bump for P = 1.0 Pa. The influence of the lower racetrack is observed with a slight bump at α = 85°, especially for P = 0.1 Pa, and to a lesser extent for P = 0.5 Pa. For P = 1.0 Pa, the distribution is broad, and the maximum is at 52° ± 32°. For the azimuthal angle, ϕ, the influence of the lateral part of the racetrack is observed, with two peaks at ϕ = −7.5 and 7.5°, especially for P = 0.1 Pa and to a lesser extent for P = 0.5 Pa. For P = 1.0 Pa, the flux is too dispersed to see this. At higher pressures (P = 0.5 and 1.0 Pa), the probability of having atoms from the whole space is observed, as shown in Figure 5.

The advantage of this representation compared to the previous one is that different conditions can be easily compared. However, the main drawback, which explains why it is not relevant for column tilt angle prediction, is as follows: first, like the previous analysis, no aggregation phenomenon is taken into account, and second, as the incidence angle α and the azimuthal angle, ϕ, distributions are plotted independently, a significant error is produced. Indeed, each graph is like a 3D map that has been folded in a single direction. For example, the large bump for the P = 1.0 Pa case is due to the sum of each small probability along the 360°. Eventually, a better incidence angle distribution would be along the ϕ = 0° direction or within a sector, e.g., ϕ = [−15, +15] or ϕ = [−30, +30]. However, this implies that a larger number of atoms is initially detected in order to maintain a sufficient number for the 2D distribution.

### 4.3. Resultant α and ϕ

As described in the previous section, the resultant incidence angle, α, and azimuthal angle, ϕ, are calculated from the resultant vector (the sum or average of each coordinate of every vector of the flux). This means that this scalar criterion is based on the aggregation of the contributions of every atom in the flux and consequently can represent growth, in contrast to the previous analyses. The drawback is that the visualization of the dispersion due to the transport is lost. However, this information is contained in the criterion. As it is based on the direction of atoms reaching a substrate obtained from simulation, complex geometrical conditions can be described (e.g., a substrate not facing the target, an object masking part of the substrates, etc.). This would not be possible with analytical models, generally speaking. Figure 4 and the four different case studies presented in Section 5 prove that this criterion is relevant for predicting the column tilt angle. Convergence tests show that for a small number of atoms (e.g., 100), the incidence angle is predicted with an accuracy lower than 5° for the highest pressure, which is the worst case of the three. If 1000 atoms are used, the accuracy is lower than 1.5°. The computation time is very short, and the sizes of the files are very small.

This subsection will explain in detail how it is built and what it represents. The 3D map analyzes the deciles used. For each decile, the resultant angles (incidence α and azimuthal ϕ) and the contribution of the decile to the total flux are calculated. The contribution is the proportion of atoms in a given decile compared to the total number of atoms in the dataset of the flux. An important point to understand is that not only the probability of an (α, ϕ) cell has to be considered but also the contribution of this cell to the flux. A second important point is that regardless of the incidence angle (small or large), the resultant incidence angle of two azimuthally opposite cells with the same contribution (small or large) will be 0°.

**Figure 7:**
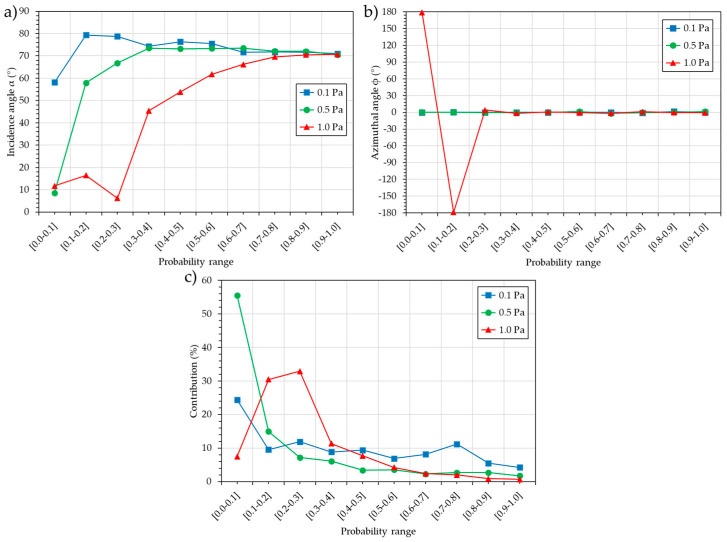
Incidence angle α (**a**), azimuthal angle ϕ (**b**), and contribution to the flux (**c**) for the ten deciles.

Figure 7 presents the incidence angle, α (a), the azimuthal angle, ϕ (b), and the contribution to the flux (c) for the ten deciles.

For P = 0.1 Pa, the incidence angle, α, except for the decile [0.0–0.1], which presents an angle of 58°, shows that all the other deciles exhibit angles between 79° and 71°, i.e., the lower and upper racetrack. The azimuthal angle, ϕ, is always equal to 0°, indicating the direction of the target. The decile [0.0–0.1], with the smallest incidence angle, α, also has the highest contribution at 24%. The seven following deciles have a contribution of around 10% each, and the last two deciles, with the highest probability, contribute 5.5 and 4.2%, respectively. Overall, 76% of the flux contributes to the growth, with an average incidence angle, α, of 75.5°. The remaining 24% contributes with an incidence angle, α, of 58°, resulting in a resultant incidence angle, α, of 71.2°.

For P = 0.5 Pa, the incidence angle, α, shows an increase from 8.5° to 66.8° for the first three deciles, while all the other deciles present angles between 73.5° and 70.5°. The azimuthal angle, ϕ, is always equal to 0°, indicating the direction of the target. As in the previous case, the decile [0.0–0.1] with the smallest incidence angle, α, also produces the highest contribution at 55.5%. The following decile [0.1–0.2], with an incidence angle of 58°, contributes 15%. The following eight deciles have a decreasing contribution from 7% to 1.7%. Overall, only 44.5% of the flux is involved in the growth, with an average incidence angle, α, of 70.3°. The remaining 55.5% contribute with an incidence angle, α, of 8.5°; the resultant incidence angle, α, is then 40°.

For P = 1.0 Pa, the first three deciles display an average incidence angle, α, of 11.5°. In the following deciles, an increase in the incidence angle, α, from 45° to 70.7° is observed. These first three deciles, with a low incidence angle, correspond to 70.8% of the flux. The following seven deciles have a decreasing contribution, ranging from 11.3% to 0.7%. More dramatically, the first two deciles, with the smallest incidence angle, α, also exhibit an azimuthal angle, ϕ, of 180°, i.e., the flux comes from the back of the substrate, directly opposite the target. This explains why the resultant incidence angle, α, is 15.5°.

**Figure 8:**
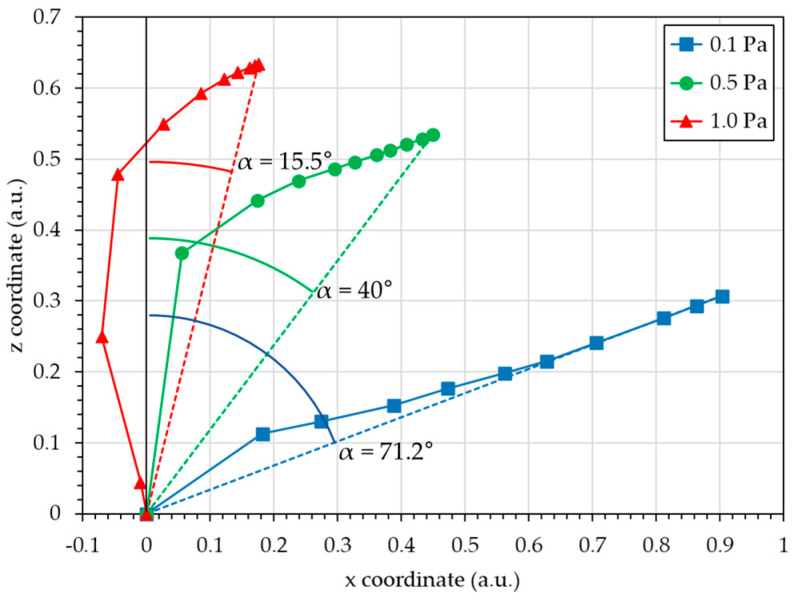
Growth vectors for the three working pressures (0.1, 0.5, and 1.0 Pa): resultant vector and decile vectors.

Figure 8 illustrates the ten vectors contributing to the flux and the resultant vectors.

This figure illustrates, in terms of vectors, the development made previously in terms of angles and contributions. Each decile vector has the direction of the incidence angle, α, and the sign of the azimuthal angle, ϕ (positive for 0° and negative for ± 180°), with a norm in proportion with the contribution.

For 0.1 Pa, the ten decile vectors are in the “+x” direction (the direction of the target) with a high inclination. Consequently, the final flux resultant vector is very similar to the ten decile vectors and presents an inclination of 71.2°. For 0.5 Pa, the first decile vector is nearly similar to the z-axis, and the nine other decile vectors are more inclined toward the “+x” direction. However, as their total norm is very similar to the first decile vector norm, the final flux resultant vector presents an inclination of 40°. For 1.0 Pa, the first three decile vectors are nearly similar to the z-axis, and more dramatically, the first two are directed toward the “−x” direction (opposite to the target direction). The seven other decile vectors are more inclined toward the “+x” direction; however, as their total norm is much smaller than that of the first three decile vectors, the final flux resultant vector presents an inclination of 15.5°.

## 5. Case Studies

In this section, four case studies are presented to illustrate the relevance of the resultant angle criterion with different process parameters: three involving a single target (working pressure, substrate position, and sputtered material) and one in co-deposition in a confocal GLAD configuration. As explained above, other contributions to growth, i.e., contamination, reactive gas, sticking coefficient, diffusion, crystallinity, material-dependent fan-out angle, substrate topography, substrate temperature, etc., which can explain the remaining deviation from the classical relationships between the incidence angle, α, and the column tilt angle, β, are not discussed, as they are outside the scope of this paper.

The experimental column tilt angle, β, is presented as a function of the experimental tilt angle, ϑ, and the calculated incidence angle, α. The Tangent rule and Tait’s rule are also plotted. The column tilt angle, β, was measured using scanning electron microscopy (SEM) analysis on the experimental fractured cross-section (not presented). The “experimental details” section presented for each case study provide the important parameters needed to reproduce the experiments in SIMTRA.

### 5.1. Working Pressure

#### 5.1.1. Experimental Details

Three argon sputtering working pressures were used: 0.11, 0.4, and 0.53 Pa. For the 0.4 and 0.53 Pa series, a 76.2 mm chromium target (purity 99.95%) was sputtered with a target-to-substrate distance of 95 mm. For the 0.11 Pa series, a 50.8 mm chromium target (purity 99.95%) was sputtered with a target-to-substrate distance of 65 mm. The substrates were centered on the target normal. The substrate tilt angle, ϑ, was increased from 0 to 85°, with a step of 5°. Each angle corresponds to a single deposition [27].

#### 5.1.2. Results

**Figure 9:**
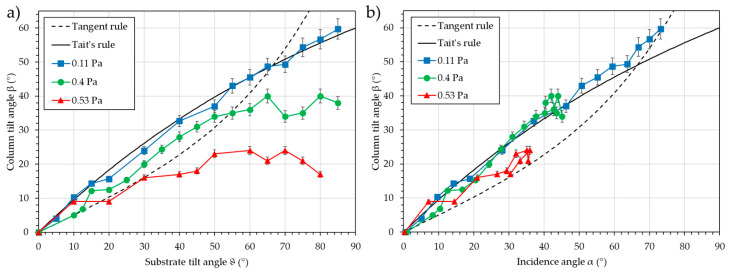
Column tilt angle, β, vs. the substrate tilt angle, ϑ (**a**), and vs. the calculated incidence angle, α (**b**) for different working pressures for chromium deposition in oblique conditions. The Tangent rule and Tait’s rule are plotted as a guide for the eye.

Figure 9 shows the column tilt angle, β, vs. the substrate tilt angles, ϑ (a), and vs. the calculated incidence angles, α (b).

As expected, the column tilt angle, β, increases continuously with the increase of the substrate tilt angle, ϑ, and the incidence angle, α. When the evolution of the column tilt angle, β, is plotted vs. the substrate tilt angle, ϑ, a leveling off is noted, with the level of the plateau depending on the pressure. The trend, except for the lowest pressure and shortest distance, does not follow any of the two classical rules. However, when the calculated incidence angle, α, is used, the three series almost overlap and closely follow Tait’s rule. This means that using this criterion along with the classical rules, it is possible to predict the column tilt angle, β, with a 5° accuracy, regardless of the pressure. Using only the substrate tilt angle, ϑ, prediction is not possible, especially when the pressure increases in combination with an increasing substrate inclination, e.g., at 0.53 Pa, the prediction is lost for substrate tilt angles ϑ greater than 30°, and at 0.4 Pa, the prediction is lost for substrate tilt angles ϑ greater than 55°.

### 5.2. Substrate Position

#### 5.2.1. Experimental Details

An argon sputtering working pressure of 0.1 Pa was used. Three transition and post-transition metals were sputtered: aluminum, chromium, and titanium (purity 99.95%). The rectangular targets had dimensions of 120 × 406 mm, and the target-to-substrate distance was 105 mm. The substrate holder’s plate was parallel to the target, and two substrate tilt angles, ϑ, were used: 0° and 85°. Five positions were set: 0 mm (aligned with the center of the target), +75 mm, +150 mm, −75 mm, and −150 mm. The 0° substrates were on the right side of the plate, all facing the target. The 85° substrates were on the left side of the plate, all facing the bottom of the chamber. A single deposition was used for each of the ten samples [28]. Another example of the influence of the substrate position can be found in previous work by Rahmouni et al. [29], which involved a circular Zr target and a radial shift of the substrates compared to the target axis.

#### 5.2.2. Results

**Figure 10:**
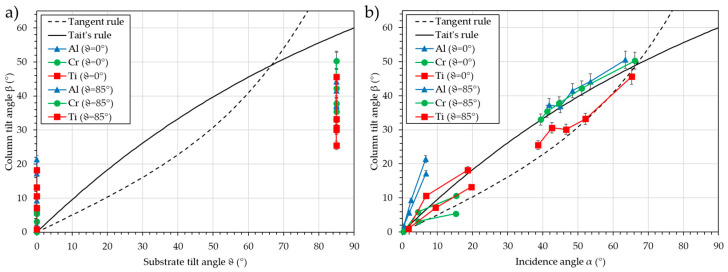
Column tilt angle, β, vs. the substrate tilt angle, ϑ (**a**), and vs. the calculated incidence angle, α (**b**) for different substrate positions for aluminum, chromium, and titanium deposition in oblique conditions. The Tangent rule and Tait’s rule are plotted as a guide for the eye.

Figure 10 presents the column tilt angle, β, vs. the substrate tilt angle, ϑ (a), and vs. the calculated incidence angle, α (b).

It can be observed that for the “ϑ = 0°” series, the column tilt angle, β, is equal to 0° at the middle position (position “0 mm”) and progressively increases to a maximum of 10° when the position changes to “+150 mm”. For the “ϑ = 85°” series, the column tilt angle, β, increases from 32° to 50° when the position varies from “+150 mm” to “−150 mm”. With the substrate tilt angles ϑ, this behavior cannot be predicted; however, using the incidence angle, α, Tait’s and Tangent rules can serve as a prediction model.

As the target is large and asymmetrical, every substrate receives a flux of matter from zones on the target with different sizes, which can be defined as “pseudo sources” [28]. The center of these “pseudo sources” moves across the target, and consequently, the geometrical substrate tilt angle changes. The second contribution is the size of these pseudo sources. For example, the substrate at “ϑ = 85°”–“+150 mm” can receive atoms from nearly the whole target surface, while the substrate at “ϑ = 85°”–“−150 mm” received atoms only from the bottom of the target, i.e., a very small surface comparatively. The asymmetry observed in the “ϑ = 0°” series is probably due to a shift in the position of the substrate holder plate, which induces a misalignment with the target.

For small substrate tilt angles, ϑ, in the case of aluminum, the calculated incidence angle, α, is underestimated. This may originate from a growth contribution or from unsuitable interaction potentials in SIMTRA for light elements.

### 5.3. Sputtered Material

#### 5.3.1. Experimental Details

An argon sputtering working pressure of 0.3 Pa was used. Nine transition metals were sputtered: titanium, vanadium, chromium, zirconium, molybdenum, hafnium, tantalum, and tungsten (purity 99.95%). Targets measuring 50.8 mm were used, with a target-to-substrate distance of 65 mm. The substrates were centered on the target normal. The substrate tilt angles, ϑ, were 0, 60, and 85°. Each angle for each material corresponds to a single deposition [30].

#### 5.3.2. Results

**Figure 11:**
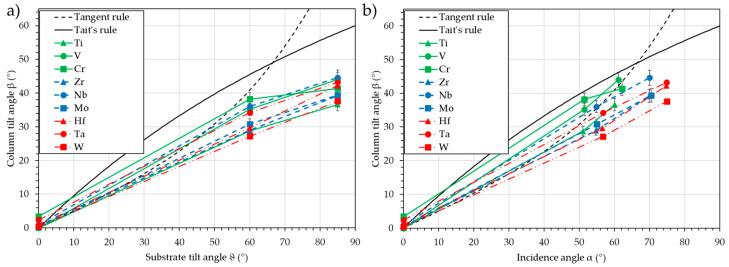
Column tilt angle, β, vs. the substrate tilt angle, ϑ (**a**), and vs. the calculated incidence angle, α (**b**) for nine transition metals deposition in oblique conditions. The Tangent rule and Tait’s rule are plotted as a guide for the eye.

Figure 11 presents the column tilt angle, β, vs. the substrate tilt angle, ϑ (a), and vs. the calculated incidence angle, α (b).

Whatever the element, and as expected, the column tilt angle, β, increases continuously with the increase of the substrate tilt angle, ϑ, and the incidence angle, α. Differences between the elements are hardly observable when the evolution of the column tilt angle, β, vs. the substrate tilt angle, ϑ, is used. Cr and the transition metals of group 5 (V, Nb, and Ta) tend to produce a higher column tilt angle, β, while the transition metals of group 4 (Ti, Zr, and Hf), Mo, and Ta show a lower column tilt angle, β. When the incidence angle, α, is used, a classification based on the period can be observed: the transition metals of period 4 (Ti, V, and Cr) have the highest column tilt angle, β, the transition metals of period 5 (Zr, Nb, and Mo) exhibit a similar column tilt angle, β, but with a higher incidence angle, α, and the transition metals of period 6 (Hf, Ta, and W) show the lowest column tilt angle, β, with the highest incidence angle, α. The prediction of the column tilt angle, β, is then possible with a 5° accuracy for the transition metals of period 4, less than 10° for period 5, and between 10 and 15° for period 6. The reason for this “period” effect is, at this stage, not clear. The interaction potential used in SIMTRA to rule the collisions may be less significant for heavy elements, or the growth phenomena may induce this decrease in the column tilt angle, β.

### 5.4. Inclined Co-Deposition

#### 5.4.1. Experimental Details

An argon sputtering working pressure of 0.3 Pa was used. Two transition metals were sputtered: chromium and tungsten (purity 99.95%). Targets measuring 50.8 mm were used, with target-to-substrate distances of 65 and 95 mm for chromium and tantalum, respectively. The substrates were centered on the intersection target’s normal. The angle between the targets was 160°, i.e., the substrate tilt angle, ϑ, is 80° for each target. The current on the targets varied from 0 to 300 mA: 300–0, 250–50, 200–100, 150–150, 100–200, 50–250, 40–260, 30–270, 20–280, 10–290, and 0–300 for chromium and tantalum, respectively. Each current configuration corresponds to a single deposition. The single chromium and tantalum depositions were simulated numerically. Based on the experimental compositions, a fraction of the flux output files was extracted and combined for each configuration. The incidence angle, α, was then calculated from these combined fluxes.

#### 5.4.2. Results

**Figure 12:**
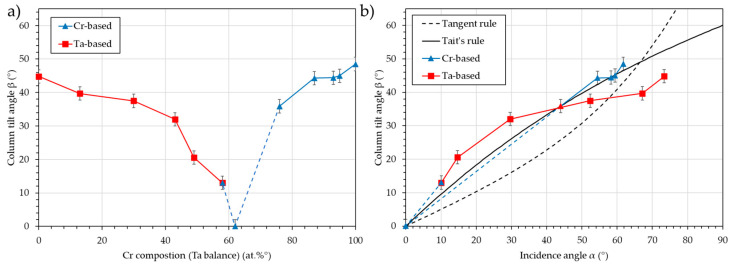
Column tilt angle, β, vs. the Cr composition (**a**) and vs. the calculated incidence angle, α (**b**) (red represents the tantalum-based films, blue represents the chromium-based films). The Tangent rule and Tait’s rule are plotted as a guide for the eye.

Figure 12 presents the column tilt angle, β, vs. the Cr composition (a) and vs. the calculated incidence angle, α (b).

The simulation of the flux in the co-deposition configuration allows for the prediction of the column tilt angle, β, using the classical rules. As noted in the previous section, this prediction is more accurate for chromium-based films than for tantalum-based films. It must be noted that in the tantalum-based films, the columns are all inclined toward the tantalum target, while in the chromium-based films, they are mostly inclined toward the chromium target. Indeed, the film with 58 at.% chromium presents a column tilt toward the tantalum target with an angle β = 13°. This can be explained by the fact that the Ta flux is more inclined than the Cr flux. A composition of 62 at.% should be used to produce films with vertically aligned columns (i.e., normal to the substrate surface). Using the resultant angle criterion, it is possible to find a substrate tilt angle, ϑ, different from the median 80°, for which the two incidence angles, α_Cr_ and α_Ta_, are equal. Then, by adjusting the current of each target, the Cr and Ta atomic flux can be balanced, and equimolar vertically aligned columns can be obtained.

## 6. Conclusions

In this paper, a comprehensive description of the sputtered fluxes was developed in terms of the direction of the atoms in order to provide reliable and predictive relationships between the incidence angle, α, and the column tilt angle, β. The different angles involved, ranging from nanometric to centimetric scales, were presented: the substrate tilt angle, ϑ, the incidence angle, α, the azimuthal angle, ϕ, the column tilt angle, β, and the crystalline tilt angle, ψ. Generally, all these angles are not equal.

To explain the three different manners of processing the direction data obtained from the transport simulation code, a typical GLAD configuration was chosen as a digital model case study, i.e., an inclined substrate with an angle ϑ = 80°, at a distance of 8 cm from a 50.8 mm planar target. Three pressures were used: 0.1, 0.5, and 1.0 Pa. The transport of the atomic flux from the target to the substrate was simulated with SIMTRA and analyzed using 3D (α, ϕ) distribution, 2D α and ϕ distributions, and resultant angles α_res_ and ϕ_res_.

The 3D (α, ϕ) distribution is useful for providing a global overview of the flux and for performing 3D growth simulations of the films. However, it cannot be used to predict the column tilt angle, β. A high number of atoms (a few 10^5^ or more) is also required.

The 2D α and ϕ distributions are useful for comparing series under different conditions. However, like the previous representation, they cannot be employed to predict the column tilt angle, β. A moderate number of atoms (a few 10^4^ or more) is also required.

In contrast, the resultant incidence angle, α_res_ (and azimuthal angle ϕ_res_), is relevant for predicting the column tilt angle, β, and only a small number of atoms (10^3^) is required. Indeed, only this criterion takes into account the aggregation phenomenon that occurs during growth.

Finally, experimental case studies were presented concerning ten elements: three hcp (hexagonal close-packing) transition metals (Ti, Zr, and Hf), six bcc (body-centered cubic) transition metals (V, Nb, Ta, Cr, Mo, and W), and one fcc (face-centered cubic) post-transition metal (Al). The influence of pressure, the position of the substrates, and the nature of the deposited element were studied in the simple OAD configuration, completed by OAD co-sputtering, which implemented two tilted and separated atomic sources. In all these cases, the resultant angle was able to predict the column tilt angle.

This powerful criterion enables the precise control of the flux and allows for detailed study of other physical contributions to growth not discussed here, namely, contamination, the combination with reactive gas, diffusion induced by temperature or energy, crystallinity, etc. It is worth noting that the criterion was successfully applied in the case of the synthesis of TiN films by reactive magnetron sputtering in an OAD configuration [31].

## Figures and Tables

**Table 1:** Summary of the different angles for the three deposition conditions.

Angle	0.1 Pa	0.5 Pa	1 Pa
Substrate tilt angle ϑ (°)	80 ± 1	80 ± 1	80 ± 1
Max incidence angle α_3D map_ (°)	70.5 ± 3.5	70.2 ± 5	70 ± 10
Max incidence angle α_2D distribution_ (°)	71.0 ± 5.5	71.0 ± 10	52.5 ± 32
Resultant incidence angle α (°)	71.2 ± 0.2	40.0 ± 1.3	15.5 ± 1.2
Column tilt angle β (°)	56.0 ± 1.9	37.4 ± 2.9	17.8 ± 1.7

## Data Availability

Data are available upon request.
